# The Protective Effect of 1,25(OH)_2_D_3_ on Myocardial Function is Mediated *via* Sirtuin 3-Regulated Fatty Acid Metabolism

**DOI:** 10.3389/fcell.2021.627135

**Published:** 2021-04-26

**Authors:** Jingxin Yang, Yalin Zhang, Yiming Pan, Can Sun, Zuwang Liu, Ning Liu, Yu Fu, Xiaofeng Li, Ye Li, Juan Kong

**Affiliations:** Department of Clinical Nutrition, Shengjing Hospital of China Medical University, Shenyang, China

**Keywords:** Vitamin D, fatty acid metabolism, Sirtuin 3, mitochondria, cardiac function

## Abstract

Energy substrate imbalance is a major cause of cardiac dysfunction. Vitamin D/vitamin D receptor (VD/VDR) deficiency is involved in the pathogenesis of various cardiac diseases; however, the exact underlying mechanism remains unclear. The aim of this study was to investigate whether vitamin D modulates mitochondrial fatty acid oxidase *via* sirtuin 3 signaling to protect the myocardium. 1-Alpha-hydroxylase-defficient mice exhibited a high metabolic rate and lower myocardial contractility than wild-type mice. Sirtuin 3 upregulation was detected in high-fat diet-fed mice receiving vitamin D3 compared with that in high-fat diet-fed mice. Both sirtuin 3 blockade and knockout inhibited the VD/VDR-induced downregulation of fatty acid oxidase in myocardial mitochondria. VD/VDR suppressed fatty acid metabolism by upregulating sirtuin 3 and lowering mitochondrial fat uptake, thereby improving myocardial function and balancing energy substrates, rather than by altering fat endocytosis and exocytosis.

## Introduction

Cardiomyocyte energy metabolism encompasses the processes of substrate absorption, distribution, and utilization. There are three main energy substrate sources in heart cells, namely glycolysis (10–20%), lactic acid (10–20%), and fatty acid oxidation (60–90%) (van Bilsen, 2004). Together, these maintain a relatively constant substrate availability in order to meet the organ’s energy demands. Heart cells adjust carbohydrate and fatty acid metabolism to adapt to various pathophysiological conditions, switching between glucose and fatty acids as the main energy source. The imbalance of energy substrates, especially the excess of fatty acids, leads to the initiation of the pathological processes in cardiac tissue, including myocardial hypertrophy and myocardial necrosis (Fillmore et al., 2014; Fukushima and Lopaschuk, 2016). Although myocardial function is known to be affected by hyperlipidemia, cardiac hypertrophy, coronary atherosclerosis, and other conditions, the detailed mechanisms underlying their effects remain unclear.

Vitamin D (VD) plays an important role in the pathogenesis of cardiovascular disease (Ku et al., 2013; Brondum-Jacobsen et al., 2012). As a liposoluble secosteroid and prohormone (Kong et al., 2013), it binds to the vitamin D receptor (VDR) (Kong et al., 2010) to regulate gene expression. VDR, a member of the nuclear receptor superfamily, plays an essential role in modulating lipid metabolism (Moya et al., 2010; Vluggens and Reddy, 2012). VD exerts its physiological effects as its bioactive form [1, 25(OH)_2_D_3_] hydroxylated by 1-alpha-hydroxylase [1α(OH)ase], which encoded by the *Cyp27b1* gene. 1,25(OH)_2_D_3_ activates the VDR to form a heterodimer complex with the retinoid X receptor, which then interacts vitamin D response elements (VDREs) in the promoter region of target genes, thus, driving the stimulation of fatty acid oxidation and the inhibition of lipogenesis by activating endogenous and exogenous SIRT1 (Sabir et al., 2017). *Cyp27b1*^–/–^ mice have vitamin D metabolism disorders and developed hypertension, cardiac hypertrophy, and cardiac contractile dysfunction (Zhang et al., 2015). Cholecalciferol cholesterol emulsion (CCE), which is composed of tea oil and VD3(93.75 μg/ml), is a safe and reliable vitamin D analog that is used in clinical practice to cure infant rickets caused by vitamin D deficiency. VDR deficiency leads to protein succinylation in white adipose tissue, providing a crucial clue regarding the underlying mechanisms of VDR in energy metabolism (Su et al., 2017).

Sirtuins are a family of nicotinamide acetylated (NAD)-dependent histone deacetylases, which are particularly sensitive to metabolic fluctuations (Lombard et al., 2007; Sadhukhan et al., 2016). Among mitochondrial sirtuins, SIRT3 appears to have the broadest deacetylase activity and is highly expressed in mitochondria-rich tissues, including muscle, liver, kidney, and heart (Lombard et al., 2007; Fukushima and Lopaschuk, 2016). In recent years, SIRT3 has been found to regulate the deacetylation of fatty acid oxidase [long-chain fatty acyl-CoA dehydrogenase (LCAD)](Hirschey et al., 2010) and electron transfer chain complexes in the mitochondria (Vassilopoulos et al., 2014). SIRT3 significantly reduces fatty acyl-coenzyme A in the tricarboxylic acid pathway as well as oxygen demand, indirectly decreasing oxygen consumption and fatty acid β-oxidation (Sack, 2012). In addition, fatty acid oxidation is upregulated in *Sirt3* knockout mice, which clearly indicates the involvement of SIRT3 in the regulation of this process (Kendrick et al., 2011). Under hyperlipidemia, the inhibition of SIRT3 and the acetylation of mitochondrial protein leads to the excessive utilization of fatty acids as energy substrates in addition to excess oxygen consumption, in turn adversely affecting cardiac cells (Lawrence et al., 1998; Sack, 2012; Fukushima and Lopaschuk, 2016). More importantly, cardiomyocyte SIRT3 expression can be inhibited by a high-fat diet (Fukushima and Lopaschuk, 2016). The deficiency of SIRT3, an anti-hypertrophic molecule, is associated with the development of cardiac hypertrophy (Sundaresan et al., 2009). However, the ability of VD to regulate SIRT3 and the exact role of the latter in VD’s effects on myocardial energy metabolism remain elusive.

To investigate the possible mechanisms underlying the cardioprotective effects of VD/VDR, we sought to link the VD/VDR signaling system with fatty acid metabolism *via* myocardial SIRT3.

## Materials and Methods

**FIGURE 1:**
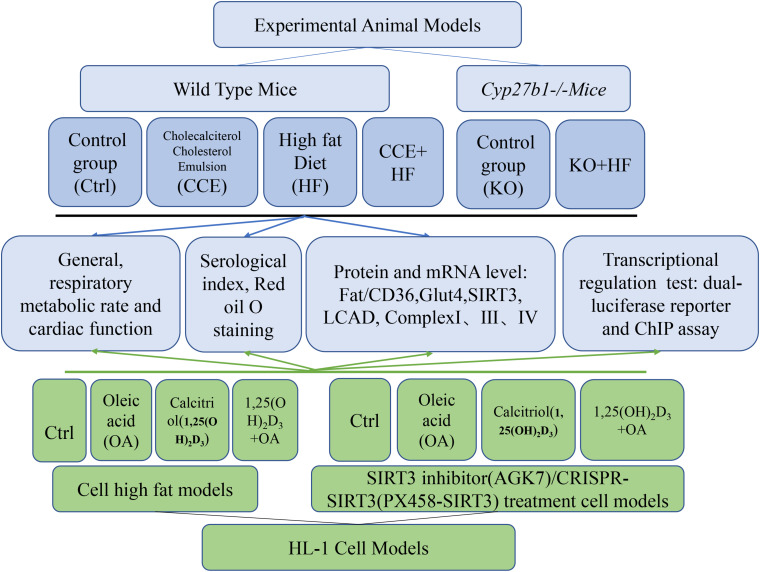
General graphical outline of the experimental design.

Our experimental process is shown in Figure 1.

### Animal Studies

ICR (Institute of Cancer Research) mice, aged 4 weeks, were purchased from Changsheng Biotechnology Co., Ltd. (Liaoning, China) and housed in the Animal Center of the Experimental Department at the Shengjing Hospital of China Medical University. Mice were maintained on a 12-h light/dark cycle and chow diet with free access to water and food. *Cyp27b1*^–/–^ mice of an ICR background were used in the current study. 1α(OH)ase is encoded by *Cyp27b1*, and its absence is characterized by VD deficiency. The knockout mice were given a high-calcium diet to maintain normal blood calcium levels. All experimental protocols in our study were approved by the Animal Experimental Committee under No. 2018PS347K. All animal procedures were carried out to minimize suffering in accordance with the guidelines established by the Animal Experimental Committee.

*Cyp27b1*^–/–^mice, originally on a BALB/c background, were repeatedly backcrossed with wild-type mice on an ICR background for 12 generations to obtain *Cyp27b1*^–/–^mice on an ICR background. *Cyp27b1*^–/–^ mice were generated through the breeding of heterozygous (*Cyp27b1*^±^) mice and identified *via* PCR-based genotyping.

WT mice were identified using the following *Cyp27b1* primer pairs:

5′-GAAGTCCCTCCTGACACAGAAACCT-3′ (forward)
and 5′-CTCATAGAGTGTCCAGGAGAGCGTA-3′ (rev erse).

The neomycin resistance gene primer pair used was 5′-ACAACAGACAATCGGCTGCTC-3′ (forward) and 5′-CTC ATAGAGTGTCCAGGAGAGCGTA-3′ (reverse).

Humans and mice are converted into body surface area equivalent doses. For people aged 14–18, the recommended dietary allowance (RDA) of VD from Institute of Medicine is 600IU/d, the daily requirement of VD given by Endocrine Society is 600-1000IU/d, and the recommended nutrient intake (RNI) of VD from Chinese Nutrition Society is 400IU/d. To sum up, take an adult who needs to intake 1000IU (25 μg) of VD per day as an example. The average body weight of adult human and mice is 70 kg and 20 g, respectively. The conversion coefficient between humans and mice is 0.026, so mice consumed dose of VD3 per day per kg weight is 25 μg/70 kg ^∗^ 70 kg ^∗^ 0.0026/20 g = 3.25 μg/kg/d.

The wild-type mice fed a normal diet were randomly divided into a control group and a group receiving CCE *via* the drinking water (CCE: water = 10 μl:100 ml) for 14 weeks (*n* = 15 in each group). In addition, wild-type mice were fed a high-fat diet (HF, 45% Cal/fat) and randomized into an HF group and a CCE + HF group (*n* = 15). The*Cyp27b1^–/–^* mice were randomly divided into a KO mice group fed a normal diet and a KO + HF group (*n* = 10) fed the high-fat diet for 14 weeks (*n* = 10). Body weight as well as food and water intake were monitored weekly. Body composition was determined using the Minispec (Bruker, LF-50). Rectal temperatures were measured at 4 p.m. using a rectal probe attached to a digital thermometer (Runjing Inc., TES-1310). All experimental mice were sacrificed under anesthesia. Blood and heart tissues were harvested and frozen for further analysis.

### Cell Culture and Treatment

The HL-1 cardiac cell line purchased from BeNa Culture Collection (BNCC, 288890) and the Cos-7 fibroblast-like cell line were cultured in DMEM with 5% glucose, 10% fetal bovine serum (FBS), and 1% penicillin/streptomycin. The cells were treated with 2 × 10^–8^ mol/l calcitriol for 2 h and subsequently challenged with 0.25 mmol/L oleic acid (OA) (Sigma-Aldrich) for 24 h.

### Biochemical Measurements

Serum triglyceride, total cholesterol, glucose, free fatty acids, calcium, and potassium were determined using commercial kits (Nanjing Jiancheng Bioengineering Institute).

### RNA Extraction and Real-Time RT-PCR

Total RNA isolated using TRIzol (Invitrogen, 15596026) was reverse-transcribed using the RT reagent kit (Takara, RR047A). Real-time PCR was conducted using SYBR Premix Ex Taq II (Takara, RR8320A). The specific primers used were synthesized by Life Co., and are listed below:

*Vdr* Forward CTGGTGACTTTGACCGGAAT;
*Vdr* Reverse CTGCACCTCCTCATCTGTGA;
*Glut4* Forward CTCTCGTCACTGACTGCACA;
*Glu4* Reverse CTCTCGTCACTGACTGCACA;
*Sirt1* Forward CGGCTACCGAGGTCCATATAC;
*Sirt1* Reverse ACAATCTGCCACAGCGTCAT;
*Sirt2* Forward AGCCGGACCGATTCAGA;
*Sirt2* Reverse TCGAGGGTCAGCTCGTCTA;
*Sirt3* Forward TTTCATGTTGGCCAAGGAGC;
*Sirt3* Reverse GCTGTTACAAAGGTCCCGTG;
*Sirt4* Forward GGGTCCTGAGCTCTTCTTGG;
*Sirt4* Reverse CCGCTCATTCTTATTCTGTCTGG;
*Sirt5* Forward CTGATGCGACCTCTCCTGAT;
*Sirt5* Reverse CCCCGAGATGATGGCTATGT;
*Sirt6* Forward ACGCAGTACGTCAGAGACAC;
*Sirt6* Reverse TCTCTCAGCTCCCCTCTACA;
*Sirt7* Forward CTACAACCGGTGGCAGGAT;
*Sirt7* Reverse AGTGACTTCCTACTGTGGCT.

### Transmission Electron Microscopy

Heart tissue samples were fixed in 2.5% glutaraldehyde with 1% paraformaldehyde, according to standard protocols. Then, 80–100 nm-thick sections were prepared.

### Western Blotting

Protein samples were isolated from tissue and cells using the Minute total protein extraction kit (Invent, SD001). Separated proteins were transferred to polyvinylidene difluoride membranes (Millipore Sigma Co., Ltd., Burlington, MA, United States). The membranes were probed with antibodies against SIRT3 (10099-1-AP, 1:500), LCAD (17526-1-AP, 1:300), Complex I (12444-1-AP, 1:1,000), Complex III (14865-1-AP, 1:1,000), Complex IV (18443-1-AP, 1:2,000), FAT/CD36 (18836-1-AP, 1:500), CPT-1β (22170-1-AP, 1:500), and GADPH (60004-1-Ig, 1:5,000) purchased from Proteintech as well as with an antibody against MTTP from Absin (abs136111a, 1:1,000) and antibodies against VDR (sc-1008, 1:1,000) and GLUT4 (sc-53566, 1:500) purchased from Santa Cruz. Following overnight primary antibody incubation, membranes were incubated with a secondary antibody (ZSGB-Bio, Beijing, China) for 1 h. The protein bands of interest were detected using Amersham ECL reagents (Cytiva, Little Chalfont, United Kingdom).

### Oil Red O Staining

Lipid accumulation in the cytoplasm was evaluated using Oil Red O staining. Myocardial tissue was formalin-fixed and frozen in Tissue Tek OCT compound (Sakura Finetek, 4,583). Then, 5 μm-thick cryosections were stained *via* Oil Red O with hematoxylin (Beyotime, C0107) used as a counterstain. Cells were fixed in 4% buffered paraformaldehyde and stained with Oil Red O and hematoxylin.

### Measurement of Cellular Oxygen Consumption Rate and Extracellular Acidification Rate

HL-1 cells were seeded in XF8 plates at a density of 60,000 cells per well to form an even monolayer, followed by treatment with OA, calcitriol, or AGK7 (SIRT3 inhibitor, Santa Cruz Biotechnology, sc-203281) for 24 h. Oxygen consumption rate (OCR) and extracellular acidification rate (ECAR) were measured in XF media under basal conditions following mitochondrial inhibitor treatment.

### Indirect Calorimetry

Mice were maintained in a comprehensive lab animal OxyletPro system (Panlab, Metabolism 3.0) through which oxygen consumption and carbon dioxide production were continuously measured for 48 h according to the instructions of the manufacturer.

### Luciferase Reporter Assays

VDREs located at nt-776 to 780 of the *Sirt3* promoter region were predicted using PROMO web. A PCR fragment with VDREs was inserted into a pGL3-basic vector confirmed by sequencing. The primers used were as follows: forward, 5′-TTCTGGTGAGAAAATTCCTGGG-3′; reverse, 5′-GACTAA CCTGGGTTACACAGCA-3′. The plasmids with VDRE and the Renilla luciferase control vector (PRL-TK) were then co-transfected into Cos-7 cells using Lipofectamine 3000 (Invitrogen, L3000-015). The luciferase activity was determined using a Dual Luciferase Assay System (Promega, e2920) with a Modulus Microplate Reader (BioTek, Synergy H1).

### Chromatin Immunoprecipitation Assay

The chromatin immunoprecipitation assay was performed using a commercial kit (Cell Signaling, 56383). Briefly, chromatin was digested and sonicated into 200–500 bp DNA fragments. The precipitated DNA was subjected to PCR analysis. The VDREs were identified using the following primer pairs: forward, 5′-CTGACAAAGCATGGGAAAGAGTG-3′ and reverse, 5′-TTGAAGGTGAGAGGGTTGAGG-3′.

### CRISPR/Cas9 System Construction and Transfection

Genome engineering was performed using a CRISPR/Cas9 system according to published protocols (Ran et al., 2013). A single guide RNA that targets the region within exon 2 of the mouse *Sirt3* was selected from genome-wide mouse sgRNA libraries. The sgRNA-optimized CRISPR design was inserted into the pSpCas9(BB)-2A-GFP vector. The primers targeting *Sirt3* were as follows: sgRNA top, 5′-TCTATACACAGAACATCGAC-3′, sgRNA bottom, 5′-GTCGATGTTCTGTGTATAGA-3′. The plasmids were then amplified, purified, sequenced, and transfected into cardiomyocytes using a jetPRIME transfection reagent (Polyplus-transfection). Western blotting was performed to confirm transfection.

### Assessment of Myocardial Function

The tracheal intubation was connected to a small animal ventilator (DW-3000B, Beijing Zhongshidichuang Co., Ltd.) under intraperitoneal anesthesia. A pressure-volume catheter was then placed in the left ventricle *via* an apical stab approach. Electrocardiogram, the maximal contractile force (Fc), and heart rate were measured using a MedLab biological signal acquisition and analysis system (JH-2, Beijing Zhongshidichuang Co., Ltd.).

### Malondialdehyde Production and Superoxide Dismutase Activity

The mice were anesthetized with ether and orbital blood was collected. The blood sample was stored at 4°C for 1 h, and then centrifuged at 3,500 *g* for 10 min, and then the serum supernatant was taken out for determination. All test kits were purchased from Nanjing Jiancheng Biological Engineering Co., Ltd., and operated in accordance with the manufacturer’s instructions. The thiobarbituric acid colorimetric method was used to determine the concentration of Malondialdehyde (MDA), and the xanthine oxidase method was used to determine the activity of superoxide dismutase (SOD).

### Statistical Analysis

Statistical analysis was performed with the Prism 8.0 software (GraphPad). An unpaired Student’s *t*-test or one-way analysis of variance followed by a Bonferroni test were used for statistical analysis after equal variance was confirmed with an F test. A p-value less than 0.05 was considered to indicate statistical significance.

## Results

### VD Regulated Energy Metabolism in High-Fat Diet-Fed Mice

**FIGURE 2:**
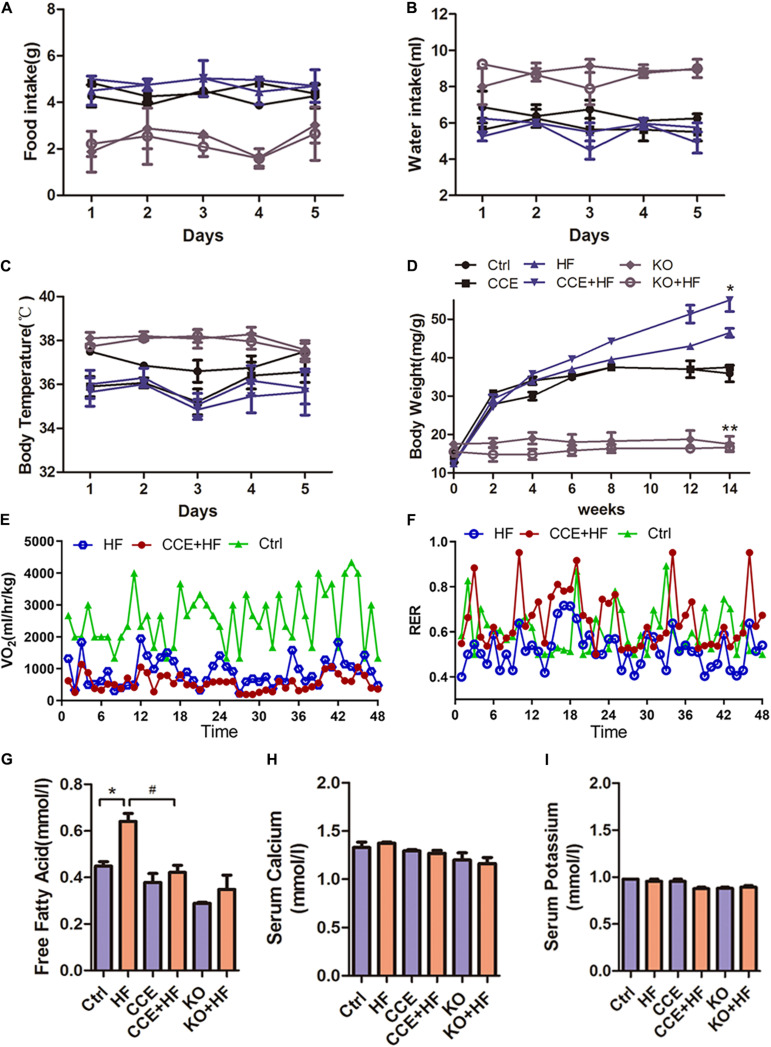
Effect of vitamin D on energy metabolism in mice fed a high-fat diet. **(A)** Body weight changes in control or KO mice receiving CCE or a high-fat diet for 14 weeks (*p < 0.05 or **p < 0.01). **(B)** Food intake, **(C)** water intake, and **(D)** body temperature in each group were monitored for 5 days, and no changes were observed in all groups (*p* > 0.05). **(E)** O_2_ consumption and **(F)** respiratory exchange rate indicated a lower metabolic rate in the CCE + HF group than in the HF group. Serum lipid levels analysis: **(G)** free fatty acids, **(H)** serum calcium, and **(I)** serum potassium, with relative changes shown for all groups (*p < 0.05 vs. Ctrl, #*p* < 0.05 vs. HF). Values are expressed as the mean ± SEM. Ctrl, control group. CCE, Fed cholecalciferol cholesterol emulsion [a precursor of 1, 25(OH)2D3] group. HF, Fed high fat die group. CCE + HF, Fed cholecalciferol cholesterol emulsion [a precursor of 1, 25(OH)2D3] and high fat die group. KO, *Cyp27b1*^–/–^ mice, KO + HF, *Cyp27b1*^–/–^ mice fed with high fat die group.

To investigate the effects of VD on energy metabolism, we examined physiological changes in each group of mice. No significant difference was observed in food intake, water intake, and body temperature among all groups (Figures 2A–C). The body weight of *Cyp27b1*^–/–^ mice was lower than that of wild-type control mice. Similarly, KO mice showed a resistance to body weight gain in response to the high-fat diet compared with that in wild-type mice. Furthermore, the highest body weight gain was observed in CCE-HF mice (Figure 2D). HF mice exhibited higher O_2_ consumption and serum free fatty acid levels (Figures 2E–G) than CCE-HF or control mice. There were no differences in serum calcium and potassium levels between all groups (Figures 2H,I), suggesting that CCE is a safe VD analog. These data indicated that VD plays a role in O_2_ consumption and lipid metabolism *in vivo*.

### VD Regulated Cardiomyocyte Fatty Acid Metabolism and Protected Against Hyperlipidemia *in vivo*

**FIGURE 3:**
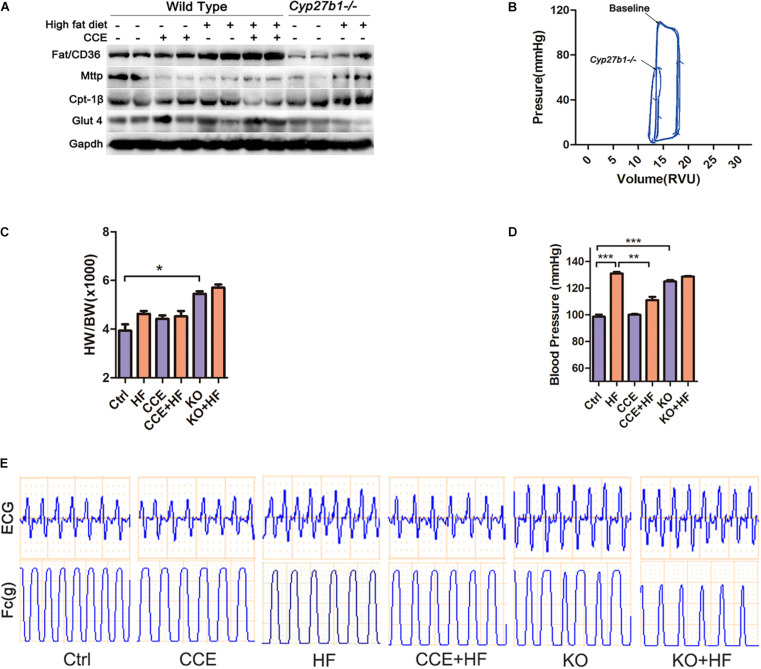
Effect of vitamin D on lipid metabolism and cardiac function *in vivo.* **(A)** Western blot of lipid metabolism-associated proteins. **(B)** The left ventricular pressure-volume loop analysis revealed that heart contractility of *Cyp27b1*^–/–^ mice was lower than the baseline. **(C)** Heart weight normalized to mouse body weight in animals fed a high-fat diet with or without CCE. **(D)** Invasive blood pressure measured *via* the carotid artery was lower in the CCE + HF group than the HF group (*p < 0.05, **p < 0.01, ***p < 0.001). **(E)** The curves of ECG and myocardial contractility were recorded for each group. Ctrl, control group. CCE, Fed cholecalciferol cholesterol emulsion [a precursor of 1, 25(OH)2D3] group. HF, Fed high fat die group. CCE + HF, Fed cholecalciferol cholesterol emulsion [a precursor of 1, 25(OH)2D3] and high fat die group. KO, *Cyp27b1*^–/–^ mice. KO + HF, *Cyp27b1*^–/–^ mice fed with high fat die group.

There were no obvious changes in the levels of endocytosis marker FAT/CD36 and exocytosis marker MTTP between HF and CCE-HF mice (Figure 3A). However, the expression of CPT-1β, a marker of mitochondrial fatty acid uptake, was significantly lower in CCE + HF mice than in HF mice, suggesting that CCE could suppress mitochondrial fatty acid utilization instead of excessive endocytosis. It is possible that VD regulates fatty acid metabolism by reducing mitochondrial fatty acid uptake and fat burning, rather than by altering fatty acid endocytosis and exocytosis. In addition, our data indicated that glucose transporter GLUT4 was upregulated in the CCE + HF group compared with that in the HF group (Figure 3A), indicating that to balance the fatty acid consumption, the body regulates glucose metabolism in the presence of VD to maintain the ratio of energy substrates. The loops of pressure-volume in *Cyp27b1*^–/–^mice hearts were much smaller than those in wild-type mouse hearts, indicative of the markedly reduced cardiac contractility in the absence of 1α(OH)ase (Figure 3B). The blood pressure and the ratio of heart weight to body weight of KO mice were higher than those of control group mice. Furthermore, the blood pressure of HF group animals was increased compared with that in control and CCE + HF mice (Figures 3C,D). Electrocardiogram and cardiac contractility analysis revealed higher, sharper R waves and worsened myocardial contractile rhythm in *Cyp27b1*^–/–^mice and HF mice than in control animals, suggesting that VD could alleviate cardiac hypertrophy and improve cardiac function (Figure 3E). Taken together, VD regulated cardiomyocyte fatty acid metabolism and protected cardiac function.

### VD/VDR Promoted the Expression of SIRT3 and Mitochondrial Respiration in the Mouse Myocardium

**FIGURE 4:**
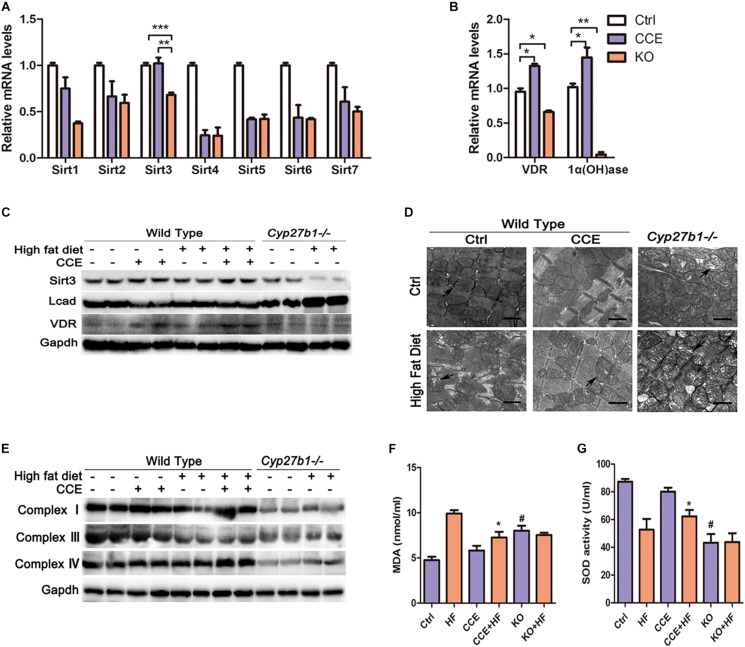
Vitamin D increased the expression of SIRT3 and mitochondrial complexes in high-fat diet-fed mice. **(A,B)** RT-qPCR analysis of sirtuins, VDR, and 1α(OH)ase revealed that the expression levels of *Sirt3*, *VDR*, and 1α(OH)ase were decreased in the KO group and increased following CCE treatment. Values are expressed as the mean ± SEM, *p < 0.05, **p ≤ 0.01, and ***p ≤ 0.001. **(C)** Western blotting analysis of SIRT3, LCAD, and VDR protein levels. **(D)** Electron microscopy images indicated mitochondrial arrangement and morphological characteristics (12,000×). Aberrant mitochondrial structures are indicated by a black arrow. **(E)** Western blotting indicated that CCE upregulated mitochondrial complex I, III, and IV protein expression. Serum levels of **(F)** MDA and **(G)** SOD with relative changes shown for the CCE + HF group compared with the HF group (#*p* < 0.05 vs. Ctrl, *p < 0.05 vs. HF). Ctrl, control group. CCE, Fed cholecalciferol cholesterol emulsion [a precursor of 1, 25(OH)2D3] group. HF, Fed high fat die group. CCE + HF, Fed cholecalciferol cholesterol emulsion [a precursor of 1, 25(OH)2D3] and high fat die group. KO, *Cyp27b1*^–/–^ mice. KO + HF, *Cyp27b1*^–/–^ mice fed with high fat die group.

To explore the mechanism through which VD reduced mitochondrial fatty acid uptake, we focused on the expression of various enzymes associated with oxidative metabolism in the mitochondria. SIRT3, VDR, and 1α(OH)ase were considerably upregulated at both the mRNA and protein levels in response to CCE treatment (Figures 4A,B) and were significantly reduced in the heart tissue of KO mice (Figure 4C) when compared with those in the heart tissue of control group animals. Consistent with previous studies (Bharathi et al., 2013), we found that a high-fat diet inhibited SIRT3 activity and protein expression, an effect that was reversed by CCE treatment. Similarly, CCE blocked the upregulated expression of LCAD caused by hyperlipidemia (Figure 4C). Transmission electron microscopy revealed severe damage of mitochondria in HF and KO mice, which CCE treatment rescued (Figure 4D). Compared with the HF group, in VD-HF animals, the protein expression levels of mitochondrial respiratory chain complexes I, III, and IV were increased (Figure 4E). Figures 4F,G indicate that VD significantly alleviated malondialdehyde (MDA) production and promoted superoxide dismutase (SOD) activity to protect cardiomyocytes against oxidative damage. Taken together, VD/VDR upregulated SIRT3 and mitochondrial respiratory chain complexes, while decreasing hyperlipidemia-induced oxidative damage.

### VD/VDR-Mediated Mitochondrial Respiration Required SIRT3 *in vitro*

**FIGURE 5:**
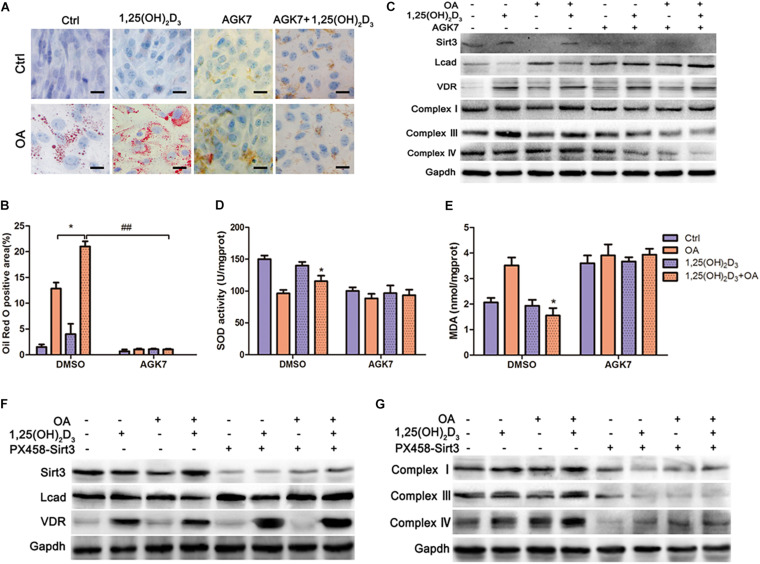
VD/VDR regulated lipid metabolism and mitochondrial respiratory metabolism *via* SIRT3 to protect HL-1 cells against oxidative damage. **(A,B)** representative Oil Red O staining in cells treated with 1,25(OH)_2_D_3_, oleic acid (OA), and AGK7 (SIRT3 inhibitor) (original magnification: 400×). *p < 0.05, ##*p* < 0.01. **(C)** Protein levels of SIRT3, LCAD, VDR, complexes I, III, and IV in HL-1 cells treated with 1,25(OH)_2_D_3_, OA, and AGK7 were analyzed *via* western blot. Analysis of **(D)** MDA and **(E)** SOD with relative changes shown for the 1,25(OH)_2_D_3_ + OA group compared with the OA group (*p < 0.05 vs. OA). **(F,G)** Western blot analysis of SIRT3, LCAD, complexes I, III and IV in cells with and/or without 1,25(OH)_2_D_3_, knockout of *Sirt3 via* CRISPR/Cas9, and OA, indicated that VD/VDR did not regulate fatty acid metabolism in the absence SIRT3.OA, oleic acid. PX458-Sirt3, knockout of Sirt3 *via* CRISPR/Cas9. AGK7, SIRT3 inhibitor.

As a SIRT3 antagonist, AGK7 cleared accumulated oil droplets in VD-HF cells, which suggested that VD might increase intracellular lipid droplets through SIRT3 (Figures 5A,B). In addition, AGK7 blocked the decrease of LCAD protein expression and the increase of mitochondrial complex expression following treatment with VD in OA (Figure 5C). However, VD was unable to reduce the expression of LCAD and upregulated mitochondrial complexes in the absence of SIRT3. We then examined the degree of oxidative damage in cells, and found that SOD and MDA levels did not change significantly after blocking SIRT3 (Figures 5D,E). Similarly, LCAD protein expression was markedly upregulated in the Sirt3^–/–^ cardiomyocytes following 1,25(OH)_2_D_3_-OA treatment relative to SIRT3-competent cells. The opposite trend was observed with regard to complex I, III and IV protein expression (Figures 5F,G). These results indicated that VD/VDR regulated mitochondrial respiratory metabolism in a SIRT3-dependent manner.

### AGK7 Partly Abolished the Effect of VD/VDR on Energy Substrate Homeostasis in HL-1 Cells

**FIGURE 6:**
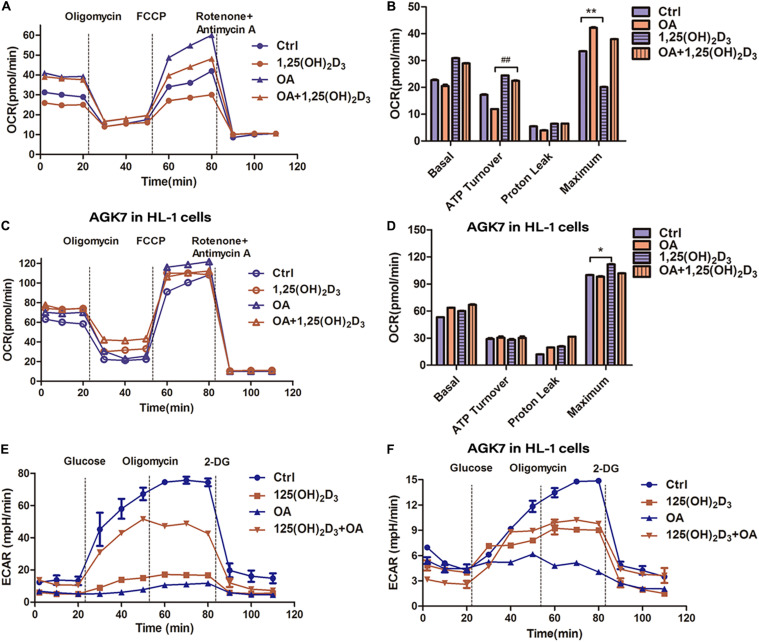
VD/VDR regulated energy substrate homeostasis in HL-1 cells *via* SIRT3. **(A)** Analysis of oxygen consumption rate (OCR) in the mitochondria of HL-1 cells treated with 1,25(OH)_2_D_3_ and OA. **(B,D)** The analysis of OCR in HL-1 cells treated with 1,25(OH)_2_D_3_, OA, and AGK7, indicating that VD treatment had no effect in the absence of SIRT3 *p < 0.05, **p < 0.01, ##*p* ≤ 0.01. **(C)** ATP turnover was determined based on the OCR prior to oligomycin minus the OCR after oligomycin, indicating that mitochondrial respiration was decreased, and ATP was increased in the 1,25(OH)_2_D_3_ + OA-treated cells compared with the OA-treated cells. **(E,F)** The analysis of extracellular acidification rate (ECAR) in HL-1 cells treated with 1,25(OH)_2_D_3_, OA, and AGK7. Ctrl, control group. OA, oleic acid. AGK7, SIRT3 inhibitor.

The oxygen consumption of mitochondria in HL-1 cells treated with 1,25(OH)_2_D_3_-OA was considerably lower than that of OA-treated cells, as determined *via* the Seahorse analyzer (Figure 6A,B). However, there was no difference in OCR and ATP production between 1,25(OH)_2_D_3_-OA- and OA-treated cells following AGK7 addition, supporting the crucial role of VD in mitochondrial metabolism *via* SIRT3. Furthermore, AGK7 stimulated high OCRs (Figures 6C,D). Interestingly, blocking SIRT3 suppressed glycolysis (Figures 6E,F), indicating that changes in a single substrate can affect overall energy homeostasis and that the maintenance of cell glycolysis by VD stabilized the proportion of energy substrates.

### VDR Binds to the VDRE to Promote *Sirt3* Expression

**FIGURE 7:**
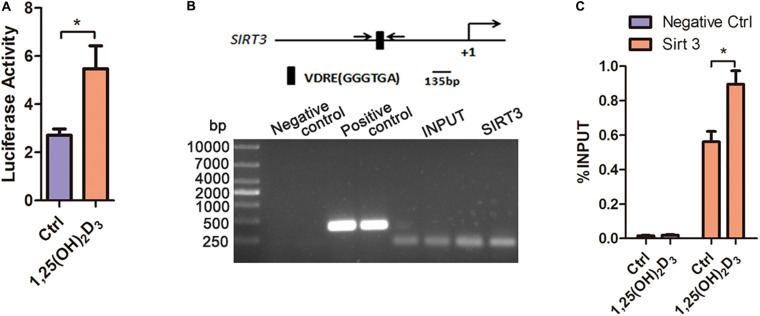
The binding of VDR to the VDRE in the promoter region of *Sirt3* increased *Sirt3* transcriptional activity. **(A)** Luciferase assay analysis indicated that 1,25(OH)_2_D_3_ increased *Sirt3* transcriptional activity, *p < 0.05. **(B,C)** PCR and RT-qPCR analysis of chromatin immunoprecipitation confirmed that VD promoted the binding of VDR to the VDRE in the *Sirt3* promoter, *p < 0.05.

The transcriptional binding sites for VDR in the *Sirt3* promoter were predicted using PROMO virtual laboratory. A twofold ratio of Firefly to Renilla was induced by calcitriol (Figure 7A). Moreover, chromatin immunoprecipitation indicated that VD promoted the binding of VDR to the SIRT3 VDRE in HL-1 cardiac myocyte cells (Figures 7B,C). These results suggested that VD increased the expression of *Sirt3* through VDR binding at the VDRE within the *Sirt3* promoter.

## Discussion

A high-fat diet intake can cause undesirable changes in cardiac structure and function. Mice on a high-fat diet will develop obesity, cardiac remodeling, contractile dysfunction, mitochondrial abnormalities, an abnormal ultrastructure, inflammation, cardiomyocyte apoptosis, etc (Ren et al., 2020; Gong et al., 2020; Zhang and Ren, 2016). Our experimental results were in agreement with some of the above (Figures 2D, 3D,E, 4D). CCE significantly improved cardiac function and myocardial contractility in mice with hyperlipidemia (Figure 3H). Interestingly, CCE markedly reduced the metabolic rate of hyperlipidemic mice, with no obvious changes in diet, water intake, and body temperature (Figure 2). Previous works have suggested a role for VDR in the regulation of energy metabolism *in vivo* (Cianferotti and Demay, 2007; Nimitphong et al., 2012). In addition, VDR^–/–^ and *Cyp27b1*^–/–^mice were reported to exhibit increased energy expenditure (Bouillon et al., 2014). Because maintaining a certain energy substrate ratio is a prerequisite for normal cardiac function, it is speculated that VD/VDR can protect cardiomyocytes *via* regulation of fatty acid utilization.

Hyperlipidemia is known to compromise cardiac function (Zittermann, 2006; Forman et al., 2007; Lee et al., 2008). However, the exact underlying mechanism remains obscure. Cardiac energy metabolite dysregulation seems to be an important factor contributing to cardiovascular disease (Fillmore et al., 2014). One of our major hypotheses is that VD can regulate the unbalanced utilization of energy substrates, thereby improving myocardial function. As expected, we observed that CCE (vitamin D3) significantly reduced lipid utilization and increased glucose uptake to balance substrates in hyperlipidemic mice (Figures 3–5). The current work provided substantial evidence regarding the VD-mediated reduction of fatty acid utilization. VD deficiency caused excessive fatty acid consumption in the heart. In contrast, VD induced an increase of free lipid droplets in cardiomyocytes *in vivo* (Figure 3A) as well as *in vitro* (Figure 5A). Owing to the increase of lipid droplets in the cytoplasm, it was presumed that VD either increased cellular fat uptake or reduced fat utilization by cells. However, unlike in the control group, VD did not change the expression of CD36 (endocytosis marker) and MTTP (exocytosis marker), suggesting that the amount of lipid inflow and outflow from cells was not changed by VD (Figure 3C). In contrast, the expression of CPT-1β, a marker of mitochondrial fatty acid uptake (Zhang et al., 2016), was significantly decreased by VD, indicating that VD regulated lipid metabolism by reducing mitochondrial fatty acid utilization rather than by regulating fat turnover *via* endo/exocytosis.

To balance lipid metabolism, VD enhanced glucose uptake in myocardial cells *via* upregulating of GLUT4 (Figure 3C). GLUT4 has a protective effect against hyperglycemia in diet-induced obese mice (Atkinson et al., 2013). Therefore, we suggest that VD induced GLUT4 to maintain the ratio between fat and glucose. Mitochondria have been established as essential organelles for the oxidation of fatty acids, with mitochondrial damage potentially leading to cardiac insufficiency (Drose et al., 2014; Fillmore et al., 2014). As expected, VD considerably decreased the mitochondrial and oxidative damage induced by hyperlipidemia (Figure 4).

SIRT3 is a well-established regulatory factor of lipid metabolism, which participates in mitochondrial deacetylation (Sundaresan et al., 2009; Sol et al., 2012). Cardiac dysfunction, interstitial fibrosis, myocardial cell apoptosis, and mitochondrial damage, accompanied by autophagy and mitochondrial inhibition in streptozotocin-induced diabetes models were exacerbated by knocking out *Sirt3*. In contrast, SIRT3 overexpression activated autophagy and the mitochondria, inhibiting mitochondrial damage as well as cardiomyocyte apoptosis, thereby protecting cardiomyocytes *in vitro* (Yu et al., 2017). However, as observed in previous studies, a high-fat diet induced an upregulated fatty acid oxidation rate and decreased SIRT3 expression. We also confirmed that VD improved cardiac function by upregulating SIRT3 (Figures 4, 5). SIRT3 regulates mitochondrial fatty acid oxidation through LCAD, a downstream target of SIRT3 (Hirschey et al., 2010; Zhao et al., 2010; Hirschey et al., 2011; Rardin et al., 2013). Furthermore, a high-fat diet or *Sirt3* knockout effectively enhanced LCAD acetylation, resulting in increased fatty acid peroxidation (Alrob et al., 2014). In particular, SIRT3 regulated mitochondrial fatty acid oxidation by inhibiting lysine 318/322 acetylation near the LCAD active site (Bharathi et al., 2013). As expected, our results revealed that VD considerably suppressed LCAD expression, presumably reducing the effects of mitochondrial lipid peroxidation (Figures 5D,E). These observations suggested that VD regulates fatty acid metabolism through the upregulation of SIRT3 to suppress LCAD in cardiomyocytes. To further confirm that VD regulated cardiac fatty acid metabolism through SIRT3, AGK7 and *Sirt3-*Cas9 plasmids were used to block SIRT3 activity and expression, respectively, in HL-1 cardiomyocytes. The Oil Red O staining and Seahorse analysis indicated that the regulation of fatty acid metabolism by VD was dependent on SIRT3 (Figure 6). Previously carried out acetylation proteomics revealed that the hyperacetylation of mitochondrial proteins was closely related to lower levels of NAD^+^, the co-substrate of mitochondrial complexes and SIRT3 (Hirschey et al., 2011). The production of ATP required mitochondrial complexes as well as cofactors, the availability of which was directly determined by intracellular NAD^+^/NADH levels (Drose et al., 2014). A defect of complex I led to a decrease in the NAD^+^/NADH ratio, which caused the hyperacetylation of mitochondrial protein in cardiac tissue (Karamanlidis et al., 2013). As CCE significantly induced complex I expression, we speculated that another mechanism through which VD regulated fatty acid metabolism may be the indirect enhancement of SIRT3 *via* complex I upregulation. This may represent another cardioprotective mechanism of VD.

The hydroxylation of vitamin D3 by 25-hydroxylase (CYP2R1 or CYP27A1) generates 25(OH)D3, which is further hydroxylated by CYP27B1 to produce biologically active 1,25 (OH)2D3. Under the action of CYP11A1, VD is converted into mono-di- and trihydroxy-D3 products, which are then further modified by CYP27B1, CYP27A1 and CYP24A1 (Slominski et al., 2012, 2014a, 2015a,b, 2017). Knockout of *Cyp27b1* can prevent mice from synthesizing active VD and lead to VD deficiency. The active form of VD3 exerts pleiotropic activity, and these different effects are mediated through interaction with VDR and/or with the retinoic acid orphan receptors (ROR)α, RORγ and aryl hydrocarbon receptor (Slominski et al., 2014b, 2018, 2020). VD regulates gene transcription through VDR activation, which then binds to the VDRE in target gene promoter regions as a heterodimer along with the retinoid X receptor (Haussler et al., 1997; Barrett et al., 2016). Therefore, we predicted and confirmed that VDR modulated *Sirt3* expression by binding to the VDRE within the *Sirt3* promoter region (Figure 7). These results further confirmed that VD regulated SIRT3 under hyperlipidemia.

**FIGURE 8:**
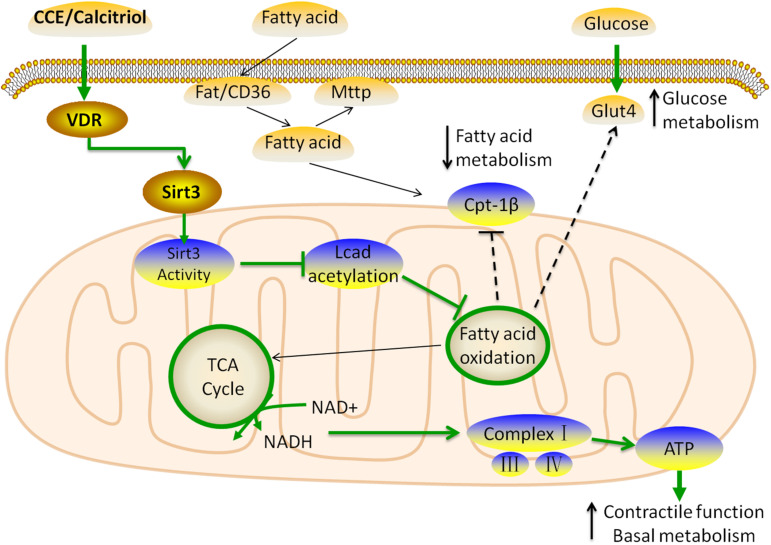
Schematic model for the role of vitamin D in the regulation of cardiac fatty acid metabolism. The VDR activated by CCE and calcitriol upregulated the expression of *Sirt3* through transcriptional regulation. SIRT3 inhibits the expression of LCAD and stimulates that of mitochondrial complexes through the use of common substrate NAD^+^, resulting in decreased fatty acid β-oxidation. VDR does not affect the inflow (Fat/CD36) and outflow (MTTP) of fatty acids, and ultimately suppresses CPT-1β to reduce the uptake of fatty acids in the mitochondria. In addition, to balance energy substrate use, VDR upregulates GLUT4, thus, promoting glucose metabolism. These changes lead to a balance of glucose and fatty acid metabolism in myocardial cells, which is beneficial to myocardial function.

In summary, we identified that the VD/VDR signaling system balanced the utilization of energy substrates to protect the heart against hyperlipidemia (Figure 8). The novel mechanisms underlying these cardioprotective effects included the following: VD/VDR (1) regulated the expression of *Sirt3* to suppress LCAD, (2) indirectly induced mitochondria complex expression *via* SIRT3 to prevent oxidative damage, (3) regulated transcriptional activity by binding to the VDRE within the *Sirt3* promoter, (4) upregulated glucose transport, and (5) suppressed mitochondrial fatty acid utilization to balance energy substrates.

## Data Availability Statement

The original contributions presented in the study are included in the article/supplementary material, further inquiries can be directed to the corresponding author/s.

## Ethics Statement

The animal study was reviewed and approved by Animal Experimental Committee of China Medical University.

## Author Contributions

JK, JY, and YZ: conceptualization. JY and YZ: methodology. CS and YP: software. JY, YZ, NL, ZL, XL, and YL: investigation. JK: resources. JY and YZ: writing—original draft preparation. JY, YZ, and JK: writing—review and editing. JK: funding acquisition. All authors have read and agreed to the published version of the manuscript.

## Conflict of Interest

The authors declare that the research was conducted in the absence of any commercial or financial relationships that could be construed as a potential conflict of interest.

## Abbreviations

VD: vitamin D
VDR: vitamin D receptor
1 α (OH)ase: 1-alpha-hydroxylase
CCE: cholecalciterol cholesterol emulsion
LCAD: long-chain fatty acyl-CoA dehydrogenase
OA: oleic acid
MDA: malondialdehyde
SOD: superoxide dismutase
OCR: oxygen consumption rate
ECAR: extracellular acidification rate
